# Necrotizing pancreatitis with invasive candidiasis and candidemia due to *Candida albicans* and pan-echinocandin-resistant *Candidaglabrata*

**DOI:** 10.1016/j.mmcr.2024.100636

**Published:** 2024-02-20

**Authors:** Laman Rahimli, Jon Salmanton-García, Philipp Kasper, Michaela Simon, Oliver A. Cornely, Jannik Stemler

**Affiliations:** a Institute of Translational Research, Cologne Excellence Cluster on Cellular Stress Responses in Aging-Associated Diseases (CECAD), University Hospital Cologne, Faculty of Medicine, University of Cologne, Cologne, Germany; b Department I of Internal Medicine, Center for Integrated Oncology Aachen Bonn Cologne Duesseldorf (CIO ABCD) and Excellence Center for Medical Mycology (ECMM), University Hospital Cologne, Faculty of Medicine), University of Cologne, Cologne, Germany; c German Centre for Infection Research (DZIF), Partner Site Bonn-Cologne, Cologne, Germany; d Department of Gastroenterology and Hepatology, Faculty of Medicine and University Hospital Cologne, University of Cologne, Cologne, Germany; e Institute for Medical Microbiology, Immunology and Hygiene, Faculty of Medicine, University Hospital of Cologne, University of Cologne, 50937, Cologne, Germany; f Clinical Trials Centre Cologne (ZKS Köln), University of Cologne, Faculty of Medicine and University Hospital Cologne, Cologne, Germany

**Keywords:** Candida albicans, Candida glabrata, Echinocandin-resistance, Necrotizing pancreatitis, Critical care

## Abstract

We report on a 64-year-old man with necrotizing pancreatitis related, invasive candidiasis, and candidemia. Despite a multidisciplinary management including antifungal therapy, endoscopic interventions and surgery, the patients’ infection progressed and lead to colon perforation, retroperitoneal abscess formation, and polymicrobial bloodstream infections. Resistance to echinocandins in *Candida glabrata* further complicated the course. This report emphasizes the need for vigilant monitoring and exploring alternative therapeutic approaches for patients in critical conditions.

## 1 Introduction

Infectious complications are a major challenge in the management of acute pancreatitis. Candida infections are common in patients with necrotizing pancreatitis with an incidence up to 39%, often leading to candidemia and were associated with high mortality [1]. Major complications of acute (necrotizing) pancreatitis includes acute necrotic collections and the development of pancreatic pseudocysts. Patients with infected pancreatic necrosis and pseudocysts have a higher risk of developing fungal infections, resulting in longer hospital stays and higher mortality rates [2]. The most prevalent species found in pancreatic necrosis and in blood cultures is *Candida albicans*, often accompanied by other *Candida* species [1]. Risk factors for pancreatic *Candida* infection include the use of antibiotics, central venous catheters, abdominal surgeries and total parenteral nutrition [3].

The presence of fungal pathogens in pancreatic infections suggests a more severe disease progression and increased comorbidity, especially among patients receiving intensive care unit treatment [2]. While fungal infections are associated with an increased mortality, the effectiveness of antifungal therapy in this population remains debated as some studies demonstrated comparable mortality rates regardless of antifungal treatment [2]. Invasive candidiasis due to echinocandin-resistant *Candida glabrata* is an emerging concern in clinical practice [4]. This phenomenon poses a significant challenge due to limited treatment options and potential adverse outcomes [4]. Here we report a patient with necrotizing pancreatitis and invasive candidiasis with candidemia due to *Candida albicans* and pan-echinocandin-resistant *Candida glabrata*.

## 2 Case presentation

A 64-year-old man with arterial hypertension, hypercholesterolemia, nicotine abuse and chronic kidney disease presented to the central emergency department (day −18) with weakness and loss of sensation in his left leg. Diagnostic work-up identified a spinal ischemia due to an aortoiliac occlusive disease (Leriche's syndrome) and the patient underwent implantation of an aorto-bi iliac prosthesis (16-8-8 mm) due to critical leg ischemia bilaterally. He was transferred to the surgical intensive care unit (ICU) for post-operative care. From day −14, the patient was transferred to a regular ward and was free of symptoms. The laboratory analysis indicated a leukocyte count of 17.04 x 10^9/L, elevated C-reactive protein levels at 265.3 mg/L, leading to the initiation of antibiotic treatment. Due to persistently elevated inflammatory parameters despite ongoing broad-spectrum antibiotic treatment with piperacillin-tazobactam, CT angiography of the abdomen was performed on day −7 to search for an infection focus, as well as to assess bowel perfusion. It showed necrotizing pancreatitis, the serum lipase concentration was 610 U/L at the admission to the hospital and reveals high levels with abdominal pain. The antibiotic therapy was changed empirically from piperacillin/tazobactam, which had been initiated 7 days ago, to meropenem. Clinical improvement resulted and inflammatory parameters decreased. Due to persistent hypokalaemia, the patient was transferred to the intermediate care ward on day −4 for further monitoring and a change of the diuretic therapy. A peripheral blood culture from day −3 showed growth of yeasts, which were identified as *Candida albicans* using matrix-assisted laser desorption ionization–time of flight mass spectrometry (Bruker Daltonik, Bremen, Germany). Antifungal susceptibility testing (AST) was performed by broth microdilution (MICRONAUT-AM, Merlin Diagnostica GmbH, Bornheim, Germany) and interpreted according to EUCAST guidelines [5]. All tested antifungal agents (anidulafungin, micafungin, fluconazole, itraconazole, voriconazole, posaconazole, amphotericin) were active. Antifungal therapy with caspofungin 70mg once a day was started on day 0 to treat *Candida albicans* candidemia due to the weight of the patient, above 80 kg. After treatment with caspofungin was initiated, the following blood cultures were sterile, and C-reactive protein and leukocyte counts decreased slightly. On day 6, follow-up abdominal sonography still showed necrotizing pancreatitis with formation of pseudocysts.

As a complication of the pancreatitis, a large retrogastric pancreatic pseudocyst developed during the further course of the inpatient stay. On day 16, this pancreatic pseudocyst was endoscopically drained into the stomach using a lumen-apposing metal stent (Hot AXIOS™ 15/10, Boston Scientific Corporation, Marlborough, Massachusetts, USA) and a double-pigtail endoprosthesis (Fig. 2). An abdominal ultrasound on day 22 continued to show necrotizing pancreatitis with regular transgastric drainage. Furthermore, there were clearly improving pseudocysts with hardly any liquid formation.

On day 27, the patient was transferred to surgical ICU for hematemesis, hemodynamic instability, and a deterioration in vigilance. After hemodynamic stabilization with vasopressors and packed red cell transfusions, a CT-guided abdominal drainage was inserted into a newly formed retrogastric fluid collection. An esophagogastroduodenoscopy with necrosectomy, and pancreatic hanarostent© (M.I Tech, Pyeongtaek-si, Gyeonggi, South Korea) placement was done on day 29. A pancreas swab grew *Candida albicans*, *Candida glabrata* and *Enterococcus faecalis*, *Escherichia coli*, *Proteus mirabilis* on day 31. One day after, another partial necrosectomy was performed during esophagogastroduodenoscopy. In addition, the position of the inserted stent was confirmed and a nasobiliary drainage placed. On the morning of day 36 a new esophagogastroduodenoscopy for necrosis ablation was performed. During the course of the procedure, the patient again developed fever. Under ongoing therapy with caspofungin, on day 38 *Candida albicans*, *Candida glabrata*, *Enterococcus faecalis*, and *Escherichia coli* were cultivated from pancreas abscess liquid and on day 42, *Candida glabrata* was also detected in blood cultures. The persistent pancreatic necrosis was clearly seen as persistent source for candidemia, however, extensive surgical resection was technically infeasible. AST of the *Candida glabrata* strain grown from blood culture during caspofungin therapy showed resistance against all echinocandins with minimal inhibitory concentrations of 0.25 mg/L (anidulafungin), 0.25 mg/L (caspofungin), 0.06 mg/L (micafungin). Therefore, antifungal therapy was changed to liposomal amphotericin B 375 mg (5 mg/kg) on day 46.

On day 52 colon perforation was suspected, and explorative surgical laparotomy performed. Descending colon perforation was detected, so that a partial resection of the transverse colon, descending colon, and sigmoid colon with Hartmann drainage was performed. Intraoperative samples from the retroperitoneal abscess revealed *Acinetobacter baumannii* (multi-resistant gram-negative bacteria, resistant to 4 different antibiotic groups (4MRGN): carbapenems, fluoroquinolones, piperacillins, and third generation cephalosporins), *Candida glabrata*, *Proteus mirabilis* and cancomycin-resistant Enterococci (VRE) *Enterococcus faecium*.

**Fig. 1:**
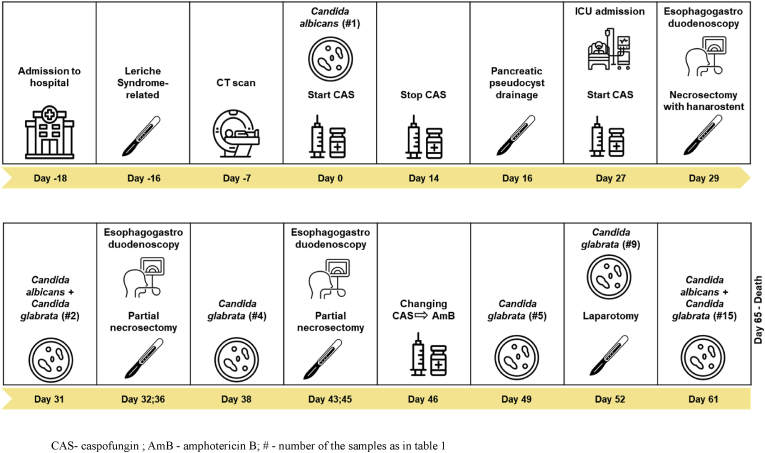
Chronology with culture results, antifungal therapy and endoscopic/surgical treatment CAS- caspofungin; AmB - amphotericin B; # - number of the samples as in Table 1.

In summary, the retroperitoneal abscess following the necrotizing pancreatitis seemed to be causative for the persistent bloodstream infections and colonic necrotizing perforation. All mycological test results are described in Table 1. Anti-infective treatment was continued with liposomal amphotericin B for evidence of caspofungin resistant *Candida glabrata*, as well as vancomycin for *Enterococcus faecium* bloodstream infection and meropenem for necrotizing pancreatitis (Fig. 1).

**Table 1:** Chronological culture results and antifungal susceptibility testing.

# sample	Type of sample	Fungal species	AST	Day
1	Blood (peripherally)	*Candida albicans*	AmB - S	0
			ANID - S	
			CAS - S	
			FLUCO - S	
			ITR - S	
			MICA - S	
			POSA - S	
			VOR - S	
2	Pancreatic juice	*Candida albicans*	–	31
		*Candida glabrata*	–	
3	Blood (CVC)	*Candida glabrata*	–	36
4	Pancreas abscess	*Candida glabrata*	–	38
5	Blood (CVC)	*Candida glabrata*	AmB - S	49
			ANID - R	
			CAS - R	
			FLUCO - I	
			MICA - R	
6	Blood (CVC)	*Candida glabrata*	–	50
7	Blood (arterial)	*Candida glabrata*	–	50
8	Blood (CVC)	*Candida glabrata*	–	51
9	Blood (Shaldon catheter)	*Candida glabrata*	–	52
10	Blood (arterial)	*Candida glabrata*	–	53
11	Blood (CVC)	*Candida glabrata*	–	53
12	Swab, wound deep abdomen	*Candida albicans*	–	57
		*Candida glabrata*	AmB - S	
			ANID - R	
			CAS - R	
			FLUCO - I	
			MICA - R	
13	Abdominal fluid	*Candida glabrata*	–	58
14	Abdominal swab	*Candida albicans*	–	61
		*Candida glabrata*	–	
15	Stent, originally placed in pancreatic cyst	*Candida albicans*	–	61
		*Candida glabrata*	–	
16	Retroperitoneal abscess, biopsy	*Candida glabrata*	AmB - S	63
			ANID - R	
			CAS - R	
			FLUCO - I	
			MICA - R	
17	Retroperitoneal perforation, abscess	*Candida glabrata*	–	63
18	Pancreas necrosis tissue	*Candida albicans*	AmB - S	64
			ANID - S	
			CAS - S	
			FLUCO - S	
			ITR - S	
			MICA - S	
			POSA - S	
			VOR - S	
		*Candida glabrata*	–	
19	Abdominal swab	*Candida albicans*	–	66
		*Candida glabrata*	–	

AmB - amphotericin B; ANID – anidulafungin; AST - antifungal susceptibility testing; CAS – caspofungin; FLUCO – fluconazole; ITR – itraconazole; MICA – micafungin; POSA – posaconazole; VOR - voriconazole

**Fig. 2:**
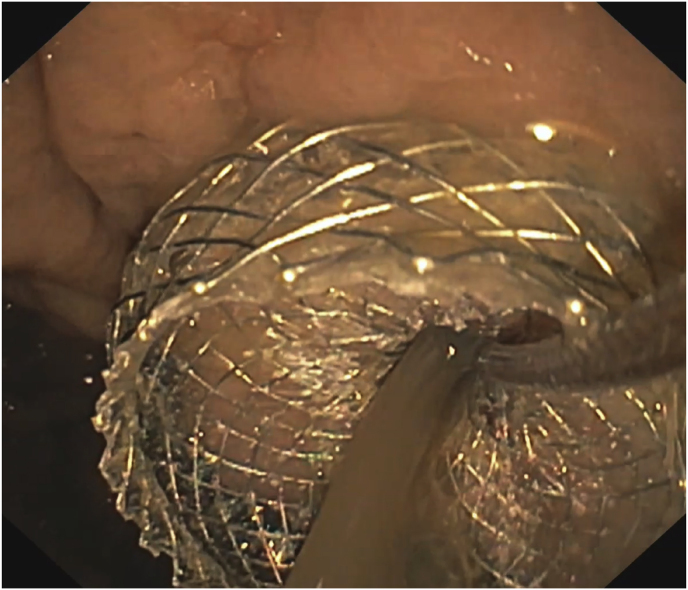
The initial drainage of the cyst with a large amount of pus. Imaging by esophagogastroduodenoscopy (on day 16)

From day 64, fever, hemodynamic instability and respiratory deterioration recurred. On the morning of day 65 a new septic episode occurred, with aspiration pneumonia and the patient was intubated. He died on day 65 due to a complicated disease course of acute necrotizing pancreatitis.

## 3 Discussion

Acute necrotizing pancreatitis is a life-threatening condition that is frequently worsened by the emergence of septic shock and multi-organ failure. The most frequently identified fungal pathogen in patients with severe acute pancreatitis is *Candida* spp. , especially *Candida albicans* [3]. Risk factors for invasive candidiasis and candidemia are listed as ICU admission, mechanical ventilation, previous antibiotic therapy, indwelling central lines, parenteral nutrition, dialysis, abdominal surgery, steroids or other immunosuppressive therapies [6].

Echinocandins and liposomal amphotericin B are the first-line medications in the initial and first-line therapy of candidiasis in both neutropenic and non-neutropenic patients, according to the European Conference on Infections in Leukemia (ECIL)-6 recommendations [7] and European Society of Clinical Microbiology and Infectious Diseases (ESCMID) Guidelines 2012 [8]. It is advised to continue treatment for 14 days beyond the initial positive blood culture. The Infectious Diseases Society of America (IDSA) and ESCMID recommend obtaining at least one blood culture every day until the results of the cultures turn negative [9]. These recommendations were followed, but unfortunately, due to deep seated infection with organ involvement, candidemia recurred. Currently there is a raising concern regarding the abovementioned 14 days of treatment continuation in candidemia cases. However, this might not be applicable to our case, as deep organs were involved too [10,11].

In the presented case, a proven *Candida* infection of pancreatic tissue was present when *Candida* spp. was isolated directly from pancreatic tissue. Treatment with caspofungin was started, venous catheter was removed, and *Candida* spp. was suspected as one of the causing pathogens for superinfected necrotizing pancreatitis with abscess formation. *Candida albicans* and *Candida glabrata* isolates were collected from the biopsy of pancreatic cysts and pancreas swabs and at the same time from blood cultures. During antifungal therapy *Candida glabrata* acquired resistance against echinocandins. As per the EQUAL *Candida* score 2018 recommendations, which is based on several international guidelines [8,12,13] and presented in more practical way for practitioners, central venous catheter (CVC) removal and echinocandin treatment was started. Daily follow-up blood cultures were checked, and caspofungin therapy was continued for at least 14 days. Echocardiography and ophthalmoscopy were not performed. To control of the quality of candidemia management, the EQUAL Score was calculated as 18 out of 22 points (82%) in this patient with an online source (https://equal.uni-koeln.de/candida/install).

*Candida* spp. are the most common pathogens of invasive fungal infections in Germany. After *Candida albicans, Candida glabrata* is found most frequently [14] and Germany is experiencing the emergence of echinocandin-resistant strains of *Candida glabrata* [15]. A study conducted in the US over a 20-year period revealed that the range of echinocandin resistance was reported to be 3.5% for *Candida glabrata*, and 0.1% for both *Candida albicans* and *Candida parapsilosis* [16]. Another extensive study involving 41 centers across 17 European countries revealed fluconazole resistance rates of 4% in *Candida tropicalis*, 12% in *Candida glabrata*, and 17% in *Candida parapsilosis*. Additionally, echinocandin-resistant cases were observed in 3% of *Candida albicans* and *Candida glabrata*, and 5% of *Candida parapsilosis* [17]. High azole and regular echinocandin usage in hospitals are typically associated with the development of resistant *Candida glabrata*. Echinocandin resistance in *Candida glabrata* is mostly related to *FKS2* gene mutations [15]. However, compared to other echinocandins, caspofungin is connected to a higher risk of stimulating *FKS2* mutations [18]. Increasing rates on drug resistant Candida infections create demand for new therapeutic approaches. Manogepix, is a novel *Gwt1* enzyme inhibitor. Its *in vitro* activity has been reported against *Candida* spp., including isolates of *Candida albicans*, *Candida auris* and *Candida glabrata* resistant to triazoles and echinocandins. Ibrexafungerp, developed as an alternative oral treatment, is a triterpenoid antifungal that inhibits the biosynthesis of 1,3-beta-D-glucan in the fungal cell wall. It has potent activity against echinocandin resistant *Candida glabrata* isolates with FKS mutations and has broad and fungicidal activity particularly against yeasts including *Candida auris* [19]. Significantly, notwithstanding the patient's final outcome, the medical team encountered no restrictions in their diagnostic and treatment capacities. Nonetheless, it is imperative to acknowledge that the generalizability of these findings may vary, and alternative methodologies may be requisite in specific contexts compared to those implemented with the aforementioned patients [20,21].

This case study reports persistent candidemia in the setting of an uncontrolled abdominal deep-seated invasive candidiasis, resulting in acquired pan-echinocandin resistance in *Candida glabrata*. Besides, the patient had several other infectious complications such as multi-drug resistant gram-negative bacteraemia with septic shock and aspiration pneumonia. Despite interdisciplinary management (critical care, endoscopy, infectious diseases, microbiology, surgery) the patient deceased.

## Ethical statement

The authors confirm that the ethical policies of the journal, as noted on the journal's author guidelines page, have been adhered to. No ethical approval was required. The relatives of the deceased patient consented to publish anonymous information on his case.

## CRediT authorship contribution statement

**Laman Rahimli:** Data collection, Writing – review & editing. **Jon Salmanton-García:** Writing – review & editing. **Philipp Kasper:** Providing the figure from gastroenterology, Writing – review & editing. **Michaela Simon:** Microbiological, Formal analysis, Writing – review & editing. **Oliver A. Cornely:** Writing – review & editing. **Jannik Stemler:** Responsible for patient treatment and follow-up, Writing – review & editing, All authors contributed to manuscript writing and review of the manuscript. All authors read and approved the manuscript.

## Declaration of Competing interest

There were no conflicts of interest to declare regarding this case report.

## References

[1] Rasch S., Mayr U., Phillip V., Schmid R.M., Huber W., Algül H., Lahmer T. (2018). Increased risk of candidemia in patients with necrotising pancreatitis infected with candida species. Pancreatology.

[2] Reuken P.A., Albig H., Rödel J., Hocke M., Will U., Stallmach A., Bruns T. (2018). Fungal infections in patients with infected pancreatic necrosis and pseudocysts: risk factors and outcome. Pancreas.

[3] Chakrabarti A., Rao P., Tarai B., Shivaprakash M.R., Wig J. (2007). Candida in acute pancreatitis. Surg. Today.

[4] Arendrup M.C., Perlin D.S. (2014). Echinocandin resistance: an emerging clinical problem?. Curr. Opin. Infect. Dis..

[5] The European Committee on Antimicrobial Susceptibility Testing (2020).

[6] Ostrosky-Zeichner L., Shoham S., Vazquez J., Reboli A., Betts R., Barron M.A. (2014). MSG-01: a randomized, double-blind, placebo-controlled trial of caspofungin prophylaxis followed by preemptive therapy for invasive candidiasis in high-risk adults in the critical care setting. Clin. Infect. Dis..

[7] Tissot F., Agrawal S., Pagano L., Petrikkos G., Groll A.H., Skiada A. (2017). ECIL-6 guidelines for the treatment of invasive candidiasis, aspergillosis and mucormycosis in leukemia and hematopoietic stem cell transplant patients. Haematologica.

[8] Cornely O.A., Bassetti M., Calandra T., Garbino J., Kullberg B.J., Lortholary O. (2012). ESCMID* guideline for the diagnosis and management of Candida diseases 2012: non-neutropenic adult patients. Clin. Microbiol. Infect..

[9] Mellinghoff S.C., Hoenigl M., Koehler P., Kumar A., Lagrou K., Lass-Flörl C. (2018). EQUAL Candida Score: an ECMM score derived from current guidelines to measure QUAlity of Clinical Candidaemia Management. Mycoses.

[10] Salmanton-García J., Reinhold I., Prattes J., Bekaan N., Koehler P., Cornely O.A. (2023). Questioning the 14-day dogma in candidemia treatment duration. Mycoses.

[11] Vena A., Bovis F., Tutino S., Santagostino Barbone A., Mezzogori L., Ponzano M. (2023). Short course of antifungal therapy in patients with uncomplicated Candida bloodstream infection: another case of less is more in the clinical setting?. Open Forum Infect. Dis..

[12] Pappas P.G., Kauffman C.A., Andes D.R., Clancy C.J., Marr K.A., Ostrosky-Zeichner L. (2016). Clinical practice guideline for the management of candidiasis: 2016 update by the infectious diseases society of America. Clin. Infect. Dis..

[13] Bow E.J., Evans G., Fuller J., Laverdière M., Rotstein C., Rennie R. (2010). Canadian clinical practice guidelines for invasive candidiasis in adults. Can. J. Infect Dis. Med. Microbiol..

[14] von Lilienfeld-Toal Mw Johannes, Einsele Hermann, Cornely Oliver A., Kurzai Oliver (2019). Invasive Pilzinfektionen: neue Herausforderungen und neue Therapieoptionen. Dtsch. Ärztebl..

[15] Aldejohann A.M., Herz M., Martin R., Walther G., Kurzai O. (2021). Emergence of resistant Candida glabrata in Germany. JAC Antimicrob Resist.

[16] Pfaller M.A., Diekema D.J., Turnidge J.D., Castanheira M., Jones R.N. (2019). Twenty years of the SENTRY antifungal surveillance program: results for Candida species from 1997–2016. Open Forum Infect. Dis..

[17] Arendrup M.C., Arikan-Akdagli S., Jørgensen K.M., Barac A., Steinmann J., Toscano C. (2023). European candidaemia is characterised by notable differential epidemiology and susceptibility pattern: results from the ECMM Candida III study. J. Infect..

[18] Shields R.K., Kline E.G., Healey K.R., Kordalewska M., Perlin D.S., Nguyen M.H., Clancy C.J. (2019). Spontaneous mutational frequency and FKS mutation rates vary by echinocandin agent against Candida glabrata. Antimicrob. Agents Chemother..

[19] Hoenigl M., Sprute R., Egger M., Arastehfar A., Cornely O.A., Krause R. (2021). The antifungal pipeline: fosmanogepix, ibrexafungerp, olorofim, opelconazole, and rezafungin. Drugs.

[20] Salmanton-Garcia J., Au W.Y., Hoenigl M., Chai L.Y.A., Badali H., Basher A. (2023). The current state of laboratory mycology in asia/pacific: a survey from the European confederation of medical mycology (ecmm) and international society for human and animal mycology (isham). Int. J. Antimicrob. Agents.

[21] Salmanton-Garcia J., Hoenigl M., Gangneux J.P., Segal E., Alastruey-Izquierdo A., Arikan Akdagli S. (2023). The current state of laboratory mycology and access to antifungal treatment in Europe: a European Confederation of Medical Mycology survey. Lancet Microbe.

